# Case of Rapidly Fatal Fever

**Published:** 1831-04-01

**Authors:** 


Case of rapidly fatal Fever. By M. Gualtier de Clanbry.

G——, a young gentleman, a native of the Isle of Bourbon, aged 18 years, and residing in one of the public academies of Paris, who was supposed to have given way to some venereal excesses, and who was most ardent in his studies, came under the care of the narrator on the 24th of May, 1829. He had passed several nights entirely without sleep, and on the morning of the above date was seized with lypothymia, and fell down insensible. On partial recovery, a rigor succeeded, and then a cold perspiration. The heart beat feebly and irregularly. In two hours after this attack, Mr. G. was seized with another and more violent rigor, followed by febrile re-action, in which the pulse got up to 130 in the minute, and the heat of skin was intense, with great headache and morbid sensibility of all the organs of sense. *Lavements, pediluvia, twenty leeches to the neck.* There was a slight amelioration of the symptoms from these means, which did not long continue. *25th May.* Same state —thirst urgent—heat intense—great anxiety—pains in the limbs—inflammation of the throat, with tumefaction of the uvula, tonsils, and palate. Twenty more leeches to the throat. 26th. No sleep last night—some delirium—fever violent—pulse 130—great and tumultuous action of the heart—respiration precipitous—sensibility of the skin extremely exalted—tongue red at the end and sides—white in the middle—symptoms of cerebral excitement. Venesection to ℥xviij.—twenty leeches to the back of the head—stimulant pediluvia—lavements. The blood shewed no marks of inflammation, and was not at all firm in its coagulum. The excitement diminished after the bleedings, and there was some tendency to diaphoresis; but in the evening there was an exacerbation of all the symptoms, more intense than ever, accompanied by distressing restlessness, and considerable delirium. 27th. In the morning there was a notable remission of the fever and all the symptoms, which promised a favourable crisis; but there were some appearances of an unfavourable nature, in the physiognomy, the state of the circulation, and the restlessness of the mind. Towards midday, symptoms of debility had acquired an alarming degree of intensity—delirium—jactitation—high state of morbid sensibility—constant discourse, in the third person, respecting his own decease, as having occurred, with lamentations on that event. He died at eleven o'clock in the evening.

*Dissection*, 16 *hours after death*. The venous system was gorged with black blood not at all coagulated, but even more fluid than during life. The vessels of the brain and its membranes were much injected; and the cerebral substance was peculiarly soft—almost creamy. The heart was flaccid and pale, containing clots of black blood. Its muscular substance was very soft and lacerable. The lungs were gorged with blood. The liver was enormously gorged with fluid and black blood. The spleen was enlarged, as were the kidneys. The mucous membrane of the bronchia and intestinal tube was highly injected, of a violet colour, pulpy, and soft. No ulceration or peculiar appearance in the mucous membrane of the small intestines. The muscular system generally presented a peculiarly pale and flabby appearance; and there were some sanguineous infiltrations between the muscular fibres in various places.

Into the various reflections of the narrator of this interesting case, we have not time to enter. He queries whether the series of morbid phenomena is to be ascribed to a primary affection of the blood itself, or of the nervous system—inclining to the latter hypothesis. We are disposed to agree with the author in his conjectures—and we seriously doubt the propriety of the depleting system in a young Indian, of exquisite sensibility, who had put his whole nervous fabric into a state of extreme exhaustion as well as excitability, by inordinate study, and by a still worse course, inordinate venereal excesses! We recommend the case to the attention of our readers.

